# The cytotoxic mechanism of epigallocatechin gallate on proliferative HaCaT keratinocytes

**DOI:** 10.1186/s12929-017-0363-7

**Published:** 2017-08-15

**Authors:** Yu-Wen Chu, Shu-Ting Liu, Ya-Lan Yang, Shih-Ming Huang, Wei-Ming Wang

**Affiliations:** 1 Graduate Institute of Medical Sciences, National Defense Medical Center, Taipei, 114 Taiwan, Republic of China; 2 Department of Pharmacy, Taichung Veterans General Hospital, Taichung, 407 Taiwan, Republic of China; 3 Department of Biochemistry, National Defense Medical Center, Taipei, 114 Taiwan, Republic of China; 4 Department of Dermatology, Tri-Service General Hospital, National Defense Medical Center, Taipei, 114 Taiwan, Republic of China

**Keywords:** Epigallocatechin-3-gallate, Keratinocytes, Proliferation, HPV, External genital warts

## Abstract

**Background:**

Epigallocatechin gallate (EGCG) is the major ingredient of sinecatechins ointment, approved for the treatment of external genital and perianal warts. However, the molecular mechanism for EGCG’s effect on warts resulting from the human papillomavirus (HPV) infection of keratinocytes is not well understood. HPV may survive in proliferative keratinocytes and may be involved in cell cycle regulation and progression. The objective of this study was to investigate the mechanism underlying EGCG’s treatment on external genital warts of HPV infection through the cultured keratinocyte cells from the HaCaT cell line.

**Methods:**

MTT and flow cytometry assays were used to measure cell viability and the cell cycle profile, with and without EGCG treatment, for HaCaT keratinocyte cells cultured in a calcium-free medium and 1.8 mM calcium which induced proliferative and differentiated keratinocytes, respectively, for 24 h. The expression levels of cytotoxic proteins and factors were evaluated with the RT-PCR and western blotting analysis.

**Results:**

EGCG influenced the proliferation stage but not the differentiation stage of keratinocytes. We suggest that apoptosis and autophagy might be the possible mechanism for the EGCG’s effect on the proliferative HaCaT cells. Furthermore, we found that EGCG reduced the protein levels of cyclin D1 and Zac1 (a zinc-finger protein which regulates apoptosis and cell cycle arrest 1) dose-dependently in proliferative as compared to differentiated keratinocytes. It also induced the expression of p21 and DEC1 (differentiated embryo-chondrocyte expressed gene 1), and promoted G1 arrest of cell cycle in proliferative keratinocytes.

**Conclusions:**

These results help clarify the mechanisms of EGCG treatment of HPV-infected keratinocytes and may contribute to new targets, such as Zac1 and DEC1 for external genital and perianal warts.

## Background

Epigallocatechin-3-gallate (EGCG), a major polyphenolic catechin found in green tea, has been reported to prevent oxidative damage in healthy cells [1]. It also functions as a chemo-preventive and antitumor agent [2–4]. EGCG has been found to reduce inflammatory reactions by inhibiting the expression or secretion of various inflammatory enzymes and cytokines in human neonatal epidermal keratinocytes [5]. Besides, studies have shown that EGCG enhances the differentiation of normal human keratinocytes and stimulates morphological changes in highly adherent flattened colonies [6]. EGCG also inhibits the proliferation of normal keratinocytes [2]. As we know, the epidermal homeostasis is maintained by the balance between proliferative and differentiated keratinocytes. The aforementioned pro-differentiation property exhibited by EGCG prompts us to speculate whether EGCG has specific cytotoxic effect upon proliferative keratinocytes.

In 2006, sinecatechins ointment (Veregen®) was approved by the US Food and Drug Administration for the treatment of external genital and perianal warts (EGWs), which result from human papillomavirus (HPV) infection [7]. The ointment contains 50% EGCG. However, despite receiving this approval a decade ago, the mechanism underlying the action of EGCG on EGWs remains unknown. It is believed that most of EGCG’s health benefits are due to its anti-oxidative activity, but it also displays poorly understood anti-proliferative and antiviral properties [8, 9]. The life cycle exhibited by HPV indicated the proliferative keratinocytes within basal epidermal compartment provide a favorable microenvironment for HPV infection. HPV may survive in proliferative keratinocytes and may be involved in cell cycle regulation and progression [10, 11]; it is likely that the facilitation of apoptosis is a key factor in the therapeutic success of EGCG in treating EGWs. However, a conclusive explanation is not yet possible due to the complexity of the cytotoxic mechanism, which can include apoptosis, DNA damage, cell cycle progression, autophagy, and related cytotoxic proteins, as well as the limited knowledge of the precise molecular targets of EGCG [2, 4]. The aim of this study was to obtain initial data using HaCaT cell line to elucidate the mechanism underlying EGCG’s cytotoxic effect on proliferative keratinocytes to get more understanding of therapeutic effect on EGWs exhibited by EGCG.

## Methods

### Cell culture and chemical

HaCaT cells were cultured in Dulbecco’s modified Eagle’s medium (DMEM) supplemented with 10% fetal bovine serum and 1% penicillin-streptomycin (#12100–046, ThermoFisher, USA). DMEM, containing high glucose, no glutamine, and no calcium, was used in the cell culture for the proliferative experiments (#21068–028). The DMEM medium containing indicated amount of calcium was adjust with 1 M calcium chloride for no calcium DMEM medium. EGCG and calcium chloride were purchased from the Sigma-Aldrich company (USA).

### Cell viability assay

The cells were seeded in 96-well culture plates and left to grow for one day. The cells were then exposed to concentrations of 0, 10, 50, 100, 150, and 200 μM EGCG, and 0, 1, 2, 3, 4, and 5 μM AG1478 in fresh specific DMEM for 24 h. 3-(4, 5 Dimethylthiazol-2-yl)-2, 5- diphenyltetrazolium bromide solution (MTT) (0.5 mg/mL in phosphate-buffered saline; PBS) was added to each well and the plate was incubated for 2 h at 37 °C. Dimethylsulfoxide (DMSO) (E. Merck, Darmstadt, Germany; 150 mL) was then added as a solubilizing agent and the absorbance at 540 nm was measured using an ELISA plate reader (Multiskan EX, Thermo, USA). As a control, cells treated with a medium containing no EGCG were set as 100% cell survival.

### Western blot analysis

Cell lysates were prepared in lysis buffer (100 mM Tris-HCl at pH 8.0, 150 mM NaCl, 0.1% sodium dodecyl sulfate (SDS), and 1% Triton X-100) at 4 °C. The extracts were separated by SDS-PAGE, transferred onto a polyvinylidine difluoride membrane (Millipore, USA), and detected using antibodies against α-actinin (ACTN) (sc-17,829; 1:10,000 dilution), HuR (sc-5261; 1:10,000 dilution), keratin 5 (sc-32,721; 1:1000 dilution), involucrin (sc-21,748; 1:1000 dilution), p16 (sc-468; 1:1000 dilution), p21 (sc-397; 1:1000 dilution), p53 (sc-126; 1:1000 dilution), cyclin D1 (sc-718; 1:1000 dilution), phosphorylation of the Ser-10 residue of the histone H3 (H3P) (sc-8656-R; 1:1000 dilution), and proliferating cell nuclear antigen (PCNA) (sc-25,280; 1:10,000 dilution) (Santa Cruz Biotechnology, USA), differentiated embryo-chondrocyte expressed gene 1 (DEC1) (A300-649A; 1:2000 dilution) (Bethyl Laboratories, USA), Zac1 (a zinc-finger protein which regulates apoptosis and cell cycle arrest 1) (ab129063; 1:2000 dilution), phosphorylation of the Ser-139 residue of the histone variant H2AX (γ-H2A.x) (ab81299; 1:1000 dilution) (Abcam, USA), beta-actin (β-actin) (#1854-S: 1:10,000 dilution) (Epitomics, USA), and poly (ADP-ribose) polymerase (PARP) (#9546; 1:1500 dilution), LC3B (#2775; 1:1500 dilution) (Cell Signaling, USA).

### Reverse transcription-polymerase chain reaction (RT-PCR)

Total RNA was isolated using the TRIsure reagent (Bioline, UK) according to the manufacturer’s instructions. One microgram of total RNA was subjected to RT using MMLV reverse transcriptase (Epicentre Biotechnologies, USA) for 60 min at 37 °C. The PCR analysis was run on a GeneAmp PCR system 9700 (Applied Biosystems, USA). The primers for the RT-PCR analysis were showed in Table 1.

**Table 1:** PCR primers were used in this study

Gene name	primer sequence (5’➔3′)	Replicon Size bp
*p53*	Forward: 5′-CTCTGACTGTACCACCATCCACTA-3′ Reverse: 5′-GAGTTCCAAGGCCTCATTCAGCTC-3′	373
*p21*	Forward: 5′-CTGAGCCGCGACTGTGATGCG-3′ Reverse: 5′-GGTCTGCCGCCGTTTTCGACC-3′	345
*p16*	Forward: 5′-GCAGCATGGAGCCTTCGGCT-3′ Reverse: 5′-TGCAGCACCACCAGCGTGTC-3′	274
*cyclin D1*	Forward: 5′-ATGGAACACCAGCTCCTGTGCTGC-3′ Reverse: 5′-TCAGATGTCCACGTCCCGCACGTCGG-3′	885
*DEC1*	Forward: 5′-GTACCCTGCCCACATGTACC-3′ Reverse: 5′-GCTTGGCCAGATACTGAAGC-3’	395
*Zac1*	Forward: 5’-TCTCACCAGTGTGCTCACTGTGAG-3′ Reverse: 5′-GGTGAGGTGATCCTTGCGCCCAA-3’	417
*GAPDH*	Forward: 5’-CTTCATTGACCTCAACTAC-3′ Reverse: 5′-GCCATCCACAGTCTTCTG-3’	463

### Fluorescence-activated cell sorting (FACS)

The FACS analysis was based on measurement of the DNA content of nuclei labeled with propidium iodide (PI). For the cell cycle evaluation, cells were treated using the procedure for the proliferation experiments, washed with ice-cold PBS, and incubated with PI solution (0.05% mg/mL in PBS, 0.1% Triton X-100, and 0.01% RNase) for 15 min at room temperature in the dark. The cells were then subjected to FACS, and cell cycle analysis was performed using a FACSCalibur flow cytometer (BD Biosciences, USA).

### Statistical analyses

Statistical values are expressed as the means ± SD of at least three independent experiments. All comparisons between groups were performed using the unpaired two-tailed t-test. Statistical significance was set at *p <* 0.05.

## Results

### EGCG affected the proliferation phase but not the differentiation phase of keratinocytes

**Fig. 1:**
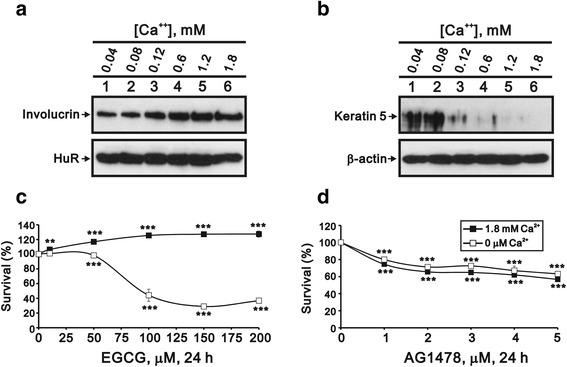
Cytotoxic effects of EGCG on HaCaT cells were greater for the proliferation stage. HaCaT cells were cultured with DMEM containing indicated amount calcium for 72 h. The proliferative and differentiation stages were examined by western blotting analysis which antibodies against (**a**) involucrin (differentiation marker) and (**b**) cytoskeleton 5 (proliferation marker) with HuR and β-actin as a control, respectively. HaCaT cells were cultured in calcium free (proliferation stage, open squares) and 1.8 mM calcium (differentiation stage, filled squares) for 24 h. The cells were subject to the cell viability assay in three independent experiments. **c** The cytotoxic effects of EGCG were only apparent in the proliferative stage. **d** In contrast, the epidermal growth factor receptor kinase inhibitor AG1478 showed similar effects with both stages. Data (**c** and **d**) are presented as mean ± SD (*n* = 3). ** *p* < 0.01; *** *p* < 0.001 versus untreated cells

The proliferation and differentiation of keratinocytes are determined by the calcium concentration in the culture medium [12, 13], with differentiation induced at higher concentrations of calcium. In our laboratory, we observed that involucrin protein expression increased and keratin 5 protein decreased with increased calcium concentration in the culture medium (Fig. 1a and b). Therefore, we used calcium-free and 1.8 mM conditions to produce proliferative and differentiated phenotype of HaCaT cells in the subsequent experiments. The cytotoxic effects of EGCG were only apparent in the proliferation stage of the HaCaT cells (Fig. 1c). In contrast, treatment with the epidermal growth factor receptor kinase inhibitor AG1478 showed similar effect for both proliferation and differentiation stages (Fig. 1d). Hence, this result suggested that the proliferation and differentiation stages of HaCaT cells have different sensitivities to EGCG.

### Cytotoxic mechanism underlying EGCG’s effect on HaCaT cells

**Fig. 2:**
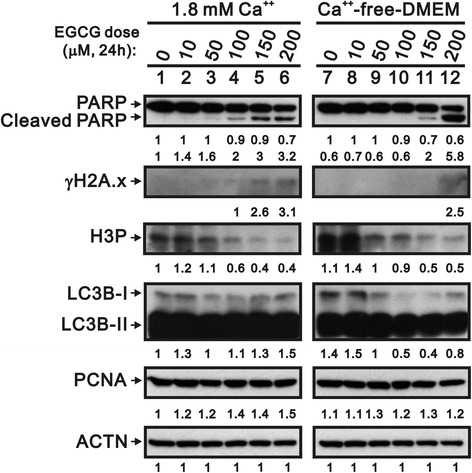
Different effects of EGCG on proteins in the two phenotypes of HaCaT cells. HaCaT cells were treated with indicated concentrations of EGCG in either calcium-free DMEM (proliferation phase) or 1.8 mM calcium (differentiation phase) for 24 h. Cell lysates were subjected to western blotting analysis and antibodies against cleaved PARP, γH2A.x, H3P, PCNA, and LC3B, with ACTN as a control. Lanes 1 to 6 are for the differentiation phase cells, and lanes 7 to 12 are for the proliferation phase cells. We then added the numerical data below each band to indicate the relative intensity of the protein in the presence of the respective concentration of EGCG, compared with lane 1 as control

Given the sensitivity to EGCG treatment of proliferative HaCaT cells, we further investigated the cytotoxic mechanism, considering apoptosis (cleaved PARP), DNA damage (γ-H2A.x), cell cycle progression (H3P), autophagy (LC3B), and proliferation (PCNA) (Fig. 2). Similar protein profiles were observed in both the proliferative and differentiated keratinocytes with H3P, PCNA, and ACTN, suggesting that these played no part in the sensitivity of proliferative HaCaT cells to EGCG. However, there were some differences between the growth stages, such as the timing and strength of the effect of EGCG on cleaved PARP, γ-H2A.x, and LC3B, especially the amount of the cleaved PARP fragment in the proliferative cells (Fig. 2, lane 12).

### EGCG reduced the expression of both cyclin D1 and Zac1

**Fig. 3:**
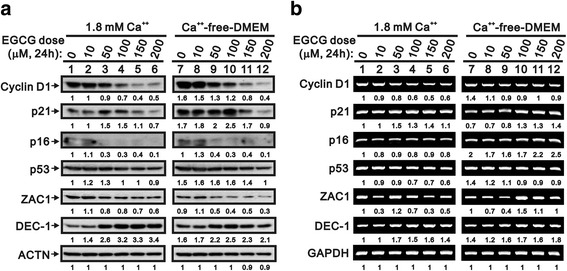
Different effects of EGCG on proteins and mRNA in the two phenotypes of HaCaT cells. HaCaT cells were treated with indicated concentrations of EGCG for 24 h and the cell lysates subjected to (**a)** Western blotting analysis and (**b)** RT-PCR analysis. We then added the numerical data below each band to indicate the relative intensity of the protein or mRNA in the presence of the respective concentration of EGCG, compared with lane 1 as control

Given the lack of an apparent difference between the proliferation and differentiation of keratinocytes in the western blotting analysis for the related cytotoxic proteins, we further examined other potential factors including cyclin D1, p21, p16, p53, Zac1, and DEC1 (Fig. 3). Before EGCG treatment, the protein expression levels of cyclin D1, p21, p53, and DEC1 were, respectively, 1.6-, 1.7-, 1.5-, and 1.6-fold higher in the proliferative than in the differentiated HaCaT cells (Fig. 3a, compare lane 7 to lane1). Similarly, the mRNA expression levels of cyclin D1, p16, p53 and DEC1 were 1.4-, 2.0-, 1.4- and 1.4-fold greater in the proliferative than in the differentiated keratinocytes respectively (Fig. 3b, compare lane 7 and lane 1). However, the mRNA expression of p21 was only 0.7-fold in the proliferative cells. Interestingly, there was no difference in the protein and mRNA expression levels of Zac1 between the two phenotype cells before EGCG treatment.

We checked the protein and mRNA level of cyclin D1 and Zac1 in response to the treatment by EGCG. The protein and mRNA expression levels of cyclin D1 of differentiated and proliferative cells were inhibited by EGCG in a dose-dependent manner (Fig. 3ab and , comparing lanes 2–6 to lane 1 and lanes 8–12 to lane 7). The Zac1 protein, not mRNA, expression was inhibited by EGCG through a dose-dependent manner in differentiated and proliferative HaCaT cells (Fig. 3a and b, comparing lanes 2–6 to lane 1 and lanes 8–12 to lane 7).

### EGCG increases the expression of p21 and DEC1 but slightly decreases p16 and p53

The p53 protein and mRNA expression was inhibited by EGCG in a dose-dependent manner, however, the slight suppression was observed at 200 μM EGCG (Fig. 3a and b, comparing lanes 2–6 to lane 1 and lanes 8–12 to lane 7). P21 is a well-known p53 target gene [14, 15]. The protein expression of p21 was enhanced by low-dose but inhibited by high-dose EGCG (Fig. 3a comparing lanes 8–12 to lane 7, comparing lanes 2–6 to lane 1). The mRNA expression of p21 was increased with EGCG in a dose-dependent manner (Fig. 3b, comparing lanes 8–12 to lane 7). Our data suggest that the expression of p21 was mediated through p53-indpendent pathway. The protein expression of another cyclin-dependent kinase (CDK) inhibitor p16 was inhibited by EGCG and its mRNA expression was enhanced slightly in 150 to 200 μM EGCG on proliferative cells (Fig. 3a and b, comparing lanes 8–12 to lane 7). DEC1, a target gene of the p53 family, mediates p53-dependent premature senescence [16]. The DEC1 protein and mRNA expression was up-regulated by EGCG in a dose-dependent manner (Fig. 3a and b, comparing lanes 2–6 to lane 1 and lanes 8–12 to lane 7). Our results suggest that the regulation of DEC1 was also mediated through a p53-indpendent pathway.

### EGCG induces G1 phase arrest in the cell cycle

**Fig. 4:**
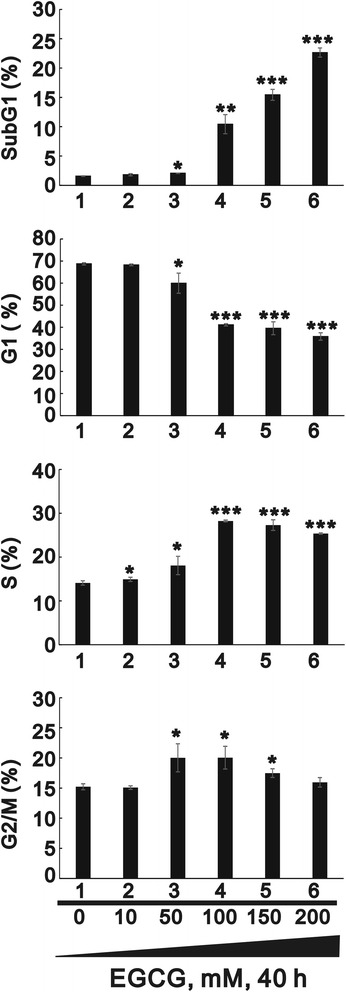
Effect of EGCG on the cell cycle profile of HaCaT cells. The differentiated HaCaT cells were treated with the indicated amount of EGCG for 40 h. Cell lysates were subjected to cell cycle profile analysis using flow cytometry in three independent experiments. Data are presented as mean ± SD (*n* = 3). * *p* < 0.05; ** *p* < 0.01; *** *p* < 0.001 versus untreated cells

We further investigated the effect of EGCG on the cell cycle profile of HaCaT cells using the flow cytometry analysis (Fig. 4). Before treatment with EGCG, the subG1 and G1 populations were lower, while the S and G2/M populations were higher in the proliferative keratinocytes. Higher concentrations of EGCG significantly induced the subG1 population and reduced the G1 population in the differentiated keratinocytes, with the S and G2/M populations increasing with EGCG in a dose-dependent manner.

## Discussion

It has been known that calcium plays an important role in the differentiation and proliferation of keratinocytes [12, 13]. Rinnerthaler et al. demonstrated that the epidermal calcium gradient of skin stimulates the proliferation of keratinocytes in the stratum basal and causes differentiation in the stratum granulosum [17]. The present study revealed an interesting finding that EGCG reduced survival rate only for the proliferative keratinocytes but AG1478 showed inhibitory effects for both proliferation and differentiation of HaCaT cells (Fig. 1), indicating ECGC inherited a proliferative keratinocyte-specific biomedical property. Because we observed expression trends for cleaved PARP, γ-H2A.x, H3P, LC3B, and PCNA in both proliferative and differentiated HaCaT cells (Fig. 2), we suggest that apoptosis and autophagy might be responsible for the effect of EGCG on the proliferative stage. The suppression of apoptotic genes has been revealed in the pathogenesis of HPV types, suggesting that the induction of apoptosis and related pathways is a major factor in the therapeutic targets of EGCG for EGWs [8, 9, 18]. Moreover, the calcium level has been reported very low within basal epidermal compartment which is the favorable microenvironment for HPV infection [7, 18]. The differential cytotoxic effect exhibited by EGCG in the presence of varied Ca2+ level indicates EGCG may eradicate the required condition for HPV infection, which also provides a plausible explanation for the therapeutic role of EGCG for EGWs [19].

It has been reported that EGCG stimulates apoptosis and induces cell cycle arrest via the regulation of apoptotic proteins and cell growth related proteins [4]. The cell cycle is closely regulated by programmed checkpoints and keratinocytic homeostasis is preserved by the equilibrium between the proliferation and differentiation of keratinocytes [20]. Cyclin D1, an activator for CDK 4/6, might regulate the progression in cell cycle [21]. In this study, the expression of cyclin D1 could be inhibited by EGCG in the dose-dependent manners especially in the proliferation-phase HaCaT cells, suggesting that EGCG might influence on the proliferation of keratinocytes through inhibiting expression of cyclin D1 (Fig. 3a and b). The activity of CDKs can be activated by cyclin D1, but is inhibited by CDK inhibitors p16 and p21 [22]. Kuo’s results showed that EGCG significantly enhanced the expression of p53 and p21, and this participated in cell cycle arrest of Hep G2 cells [15]. EGCG also causes apoptosis by p53- and Fas-mediated pathways in cancer cells [15, 23]. DEC1, is one of the effectors downstream of p53 to induce G1 arrest and premature senescence in p53- and p21-independent manners [16]. DEC1 also regulates p53-dependent DNA damage-induced apoptosis through macrophage inhibitory cytokine-1 [24]. In this study, our findings failed to support the functional role of p53 and p16 induced by EGCG, however, and the regulation of p21 and DEC1 mediated through the p53-independent pathway (Fig. 2). The detailed cell-context issue remains to be investigated in the future.

Zac1, a zinc-finger protein which regulates apoptosis and cell cycle arrest 1, or called as PLAGL1 (pleomorphic adenoma gene-like 1 gene), classified as the PLAG superfamily with a seven N-terminal copies of the C_2_H_2_-type zinc-finger motif, might play as a transcriptional factor or cofactor for nuclear receptors, p53, and HPV E2 protein [25–29]. We have previously found that Zac1 enhances the expression of cyclin D1 and may promote the cell cycle progression. These findings indicated that Zac1 plays a dual role, modulating not only cell differentiation and apoptosis but also cell proliferation [30, 31]. In previous experiments, we evidenced that curcumin inhibited Zac1-enhanced cyclin D1 expression in human keratinocytes and prevented the keratinocytes from entering the S phase in the cell cycle [26]. We also demonstrated that Zac1 enhanced the activator protein-1 (AP-1) transcriptional activity in human HaCaT keratinocytes [32]. Interestingly, the results of the present study indicated that EGCG also inhibited the expression of both Zac1 and cyclin D1. EGCG may affect expression of transcription factor AP-1 in keratinocytes [4, 33, 34]. However, a drawback of the present study is that we did not verify the dependency of AP-1 on EGCG in the aforementioned proposal. Further investigation is needed to determine whether Zac1 enhances the expression of cyclin D1 and the AP-1 transcriptional activity and whether the inhibitory mechanism of EGCG is similar to that of curcumin.

Recent studies illustrated that EGCG precipitated cell growth arrest at the G1 phase of the cell cycle through regulation of cyclin D1, CDK4, CDK6, and p21, and induced cellular apoptosis through the production of reactive oxygen species and activation of caspase-3 [15, 23, 35]. The results of the current study supported this, showing that EGCG induced G1 arrest of the differentiated keratinocytes (Fig. 4). EGCG is a multifunctional agent and has long been studied for clinical application in human diseases, including HPV infection. It exhibits an anti-proliferative property by regulating multiple cellular functional pathways that involve proliferation, survival, and various protein kinase pathways, among others.

## Conclusions

By virtue of EGCG, we found the differential response in the proliferative and differentiated HaCaT cells. In addition, the therapeutic mechanism of EGCG related cytotoxicity upon EGWs was not very clear. We suggest that apoptosis and autophagy might be the potential mechanism for the EGCG’s effect. Interestingly, our results indicated that EGCG suppressed dose-dependently cyclin D1 and Zac1 proteins in proliferation phase of keratinocytes as compared to differentiation phase. Consistent with previous studies, our results showed that EGCG stimulated the expression of p21 and DEC1, and induced the G1 arrest of keratinocytes. Here, we found that Zac1 and DEC1 might be the novel target gene of EGCG. The findings of this study shed light on the possible mechanisms by which EGCG treats external genital and perianal warts in HPV infection keratinocytes. It is hoped that, through the modulation of the important factors revealed here, this might contribute to new target therapies for disease management.

## Abbreviations

ACTN: α-actinin
AP-1: Activator protein-1
CDK: Cyclin-dependent kinase
DEC1: Differentiated embryo-chondrocyte expressed gene 1
DMEM: Dulbecco’s modified Eagle’s medium
DMSO: Dimethylsulfoxide
EGCG: Epigallocatechin-3-gallate
EGWs: External genital and perianal warts
FACS: Fluorescence-activated cell sorting
H3P: Phosphorylation of the Ser-10 residue of the histone H3
HPV: Human papillomavirus
LC3B: Light chain 3B
MTT: 3-(4,5 Dimethylthiazol-2-yl)-2,5-diphenyltetrazolium
PARP: Poly (ADP-ribose) polymerase
PBS: Phosphate-buffered saline
PCNA: Proliferating cell nuclear antigen
PI: Propidium iodide
PLAGL1: Pleomorphic adenoma gene-like 1 gene
RT-PCR: Reverse transcription- polymerase chain reaction
SDS: Sodium dodecyl sulfate
Zac1: A zinc-finger protein which regulates apoptosis and cell cycle arrest 1
γ-H2A.x: Phosphorylation of the Ser-139 residue of the histone variant H2AX
